# Topographical and Optical Characteristics of Thermoplastic Dental Appliances Materials Related to Water Sorption

**DOI:** 10.3390/jfb14040190

**Published:** 2023-03-28

**Authors:** Liliana Porojan, Flavia Roxana Toma, Mihaela Ionela Bîrdeanu, Roxana Diana Vasiliu, Anamaria Matichescu

**Affiliations:** 1 Department of Dental Prostheses Technology (Dental Technology), Center for Advanced Technologies in Dental Prosthodontics, Faculty of Dental Medicine, Victor Babes University of Medicine and Pharmacy Timisoara, Eftimie Murgu Square No. 2, 300041 Timisoara, Romania; 2 National Institute for Research and Development in Electrochemistry and Condensed Matter, A. Păunescu Podeanu Str. No. 144, 300569 Timisoara, Romania; 3 Department of Preventive, Community Dentistry and Oral Health, Center for Advanced Technologies in Dental Prosthodontics, Faculty of Dental Medicine, Victor Babes University of Medicine and Pharmacy Timisoara, Eftimie Murgu Square No. 2, 300041 Timisoara, Romania

**Keywords:** thermoplastic PET-G materials, surface topography, optical characteristics, water sorption

## Abstract

Clear thermoplastic materials have increased in popularity in the dental field due to their various applications, combination of excellent aesthetics, and good biomechanical behavior, but they may be influenced by different environmental conditions. The purpose of the present study was to assess the topographical and optical characteristics of thermoplastic dental appliances materials relative to water sorption. PET-G polyester thermoplastic materials were evaluated in this study. Related to water uptake and desiccation stages, surface roughness was analyzed, and three-dimensional AFM profiles were generated for nano-roughness measurements. Optical CIE L*a*b* coordinates were recorded and parameters like translucency (TP), contrast ratio for the opacity (CR), and opalescence (OP) were derived. Levels of color changes were achieved. Statistical analyses were performed. Water uptake significantly increases the specific weight of the materials, and after desiccation, the mass decreases. Roughness increased after water immersion as well. Regression coefficients indicated a positive correlation between TP and a* and between OP and b*. Studied PET-G materials have a different behavior to water exposure, but for all their specific weight, they increased significantly within the first 12 h. It is accompanied by an increase in the roughness values, even if they continue to be kept below the critical mean surface roughness. On nano-level, 3D images show an increase in inhomogeneity in the network structure of particles. Slight color changes were registered.

## 1. Introduction

Because of the combination of excellent aesthetics, translucency, good mechanical strength, flexibility, and various applications in the dental field, clear thermoplastic materials have increased in popularity. Vacuum formed appliances are suitable for orthodontic retainers, temporomandibular joint and periodontal splints, mouth guards, and removable tooth aligners [1,2,3].

Increased aesthetic requirements direct us more and more towards various types of thermoplastic polymers. Besides their optical characteristics, the appliances offer comfort to the patients, growing the use of this type of materials in modern dentistry [4,5].

They can be achieved from various types of thermoplastic polymers. The most commonly used polymers are polyethylene terephthalate-glycol (PET-G), polyethylene terephthalate (PET), polyethylene vinyl acetate (PEVA), thermoplastic polyurethanes (TPUs), polycarbonate (PC), polypropylene (PP), and polymers blends.

Thermoplastic polymers are classified in amorphous and semicrystalline polymers depending on their molecular structure. The first ones are characterized by an irregular molecular structure with a low degree of molecular assembling. The last ones contain both crystalline (uniform) and amorphous (irregular regions). The crystalline areas confer hardness and rigidity to the thermoplastic material. Semicrystalline polymers have mechanical strength, are hard, can be opaque or translucent, and possess good chemical resistance. Amorphous polymers are softer, transparent, are therefore more aesthetic, and have better impact resistance [6,7].

Among polyesters, polyethylene terephthalate (PET) and polyethylene terephthalate glycol (PET-G), a non-crystallizing amorphous copolymer of PET, are promising and widely used in dental field due to their excellent mechanical and optical properties. PET-G is a non-crystalline co-polyester composed of 1,4-cyclohexane two methanol (CHDM), ethylene glycol (EG), and terephthalic acid (TPA). PET-G shows very good transparency, adequate formability, shape-memory, and chemical resistance against various solvents. It can either be processed by vacuum thermoforming, computerized cut, or printed. Thus PET-G materials are transparent, aesthetic, and very durable and possess high impact strength, high fatigues resistance, three-dimensional stability, and resistance to environmental chemical changes [8,9,10,11,12,13,14,15,16,17].

The biomechanical behavior is influenced by the chemical structure of the material, the materials handling conditions, morphology, thickness, quality, and finishing technique [18]. In the oral environment the material is subject to a natural hydrothermal aging and mechanical fatigue [12]. Therefore, appliances should be replaced every 2 weeks on average for an orthodontic treatment, maintaining adequate strength. Retainers are indicated for a long-term use in order to avoid orthodontic relapse [19]. Disadvantages associated with the long-term use of aesthetic retainers include decreased translucency and integrity of the material, discoloration, and increased roughness [3,20]. Stresses induced in thermoplastic materials by deformations tend to deteriorate the mechanical properties in time. The mechanical properties of thermoplastic materials are both material and environmental dependent. For molding the thermoplastic material, provided discs must be heated and vacuum-molded in a vacuum forming equipment under appropriate processing conditions of temperature and time, avoiding failures, such as blister formation, and thermal degradation [4,21]. Knowing the physicochemical and mechanical behavior of the processed thermoplastic materials is very important.

The purpose of the present study was to assess the topographical and optical characteristics of thermoplastic dental appliances materials relative to water sorption. The null hypothesis is that topographical characteristics of the materials are influenced by water sorption, and the second that the water sorption affects the optical properties of the materials. The third null hypothesis is that there is no significant interaction between the materials taken into the study and the stage related to water sorption.

## 2. Materials and Methods

### 2.1. Specimen Preparations

Sheets of the thermoplastic materials were thermoformed with a pressure molding machine device (MINISTAR, Scheu Dental, Iserlohn, Germany). The following PET-G polyester thermoplastic materials were evaluated in this study: Crystal^®^ (Bio Art Dental Equipment, Sao Carlos, Brazil) [C], Duran^®^ (Scheu-Dental GmbH, Iserlohn, Germany) [D], Erkodur (Erkodent, Pfalzgrafenweiler, Germany) [E], and Leone (Leone SpA, Firenze, Italy) [L], with 1.0 mm thickness.

The sheets were heated at 220 °C for 30 s and pressed at 4.7 bar and vacuumed over a gypsum mold with surfaces of 10 mm × 40 mm positioned at an angle of 45°. The sheets that resulted after thermoforming were removed, and specimens were cut out and used for analyses. The thermoformed sheets were cut into square pieces with standard dimensions of 10 mm × 10 mm. Models were constructed to mimic the average dimensions and position of natural teeth. The cooling time lasted 60 s. The thickness varied depending on the material, related to thermoforming.

A total of 120 pieces (30 from each thermoplastic material) were prepared. The samples were stored in glass recipients containing distilled water or silica gel inside an incubator at 37 °C throughout the study.

### 2.2. Water Sorption and Water Solubility

For all four materials, tests for water sorption and solubility were performed according to ISO 20795-2 [22]. Specimens from each brand were dried in a desiccator at 37 °C, containing silica gel. The specimens were weighted on an analytical balance Kern ABT 100–5 NM (KERN & SOHN GmbH, Balingen, Germany), accurate to 0.00001 g. The drying and weighting cycles were repeated until a constant mass, called conditioned mass (m_1_), was reached. The specimens were then immersed in glass recipients containing distilled water inside the incubator at 37 °C for 14 days. The specimens were taken away from water after 12 h, dried of traces of water, and weighed within 60 s after removal. The specimens were re-immersed in water and the procedures of measurement repeated after at 24 h, 48 h, 72 h, 7 days, and 14 days when a constant mass, called water saturation (m_2_), was reached. The specimens were then taken away from the water and replaced in the desiccator. The desiccation process used above was repeated until constant mass, called reconditioned mass (m_3_), was reached.

The water sorption, W_sp_, and water solubility, W_sl_, are expressed in mg/mm^3^ using the Equations (1) and (2):

W_sp_ = (m_2_ − m_3_)/V (mg/mm^3^) (1)

W_sl_ = (m_1_ − m_3_)/V (mg/mm^3^) (2)

where m_1_ is the constant mass dry samples, m_2_ is the constant mass wet samples, and m_3_ is the constant mass reconditioned samples, all values in mg. V is the volume of specimens in mm^3^ [22].

### 2.3. Surface Roughness Measurements

Surface roughness was analyzed using a contact 2 µm stylus profilometer Surftest SJ-201 (Mitutoyo, Kawasaki, Japan), a sampling length of 0.8 mm, and a force of 0.7 mN. Arithmetic average roughness (Ra) and maximum absolute vertical roughness (Rz) measurements were performed for the specimens in all stages for each group, each material, and each stage. Measurements were recorded in 5 different directions. The mean value of the five measurements was calculated for each surface. 

### 2.4. Nanosurface Topographic Characterization by Atomic Force Microscopy (AFM)

Samples from each group were randomly chosen and evaluated with an atomic force microscope Nanosurf Easy Scan 2 Advanced Research (NanosurfAG, Liestal, Switzerland), in non-contact mode and values for average nano-roughness Sa (nm), maximum amplitude of heights Sy (nm), and particle size were registered. AFM generated corresponding three-dimensional profile images and color maps of the sample surfaces (4.59 µm × 4.59 µm). Measurements were made on specimens of each group, for all materials, and in all stages related to water immersion.

### 2.5. Optical and Colour Change Measurements

Optical CIE L*a*b* coordinates were recorded for each stage under D65 standard illumination using a spectrophotometer Easyshade IV (Vita Zahnfabrik, Bad Säckingen, Germany) on the black (b) and white (w) background of the grey card WhiBal G7 (White Balance Pocket Card). The device was calibrated before each measurement, and the probe tip was held at 90° to the surface of the sample. The recording was assumed when two consecutive identical readings were achieved. The measurements were performed by the same operator.

L* is the lightness coordinate (L* = 0 perfect black, L* = 100 perfect white); a* = is the chromatic coordinate in the red (positive value)/green (negative value) axis, and b* = is the chromatic coordinate in the yellow (positive value)/blue (negative value) axis [23].

Parameters like translucency (TP), opalescence (OP), and contrast ratio for the opacity (CR) were calculated according to the Formulas (3)–(5).

TP = [(L*_b_ − L*_w_)^2^ + (a*_b_ − a*_w_)^2^ + (b*_b_ − b*_w_)^2^]^1/2^ (3)

TP values may range from 0 (totally opaque) to 100 (totally transparent).

OP values estimate the difference in red–green and yellow–blue colour coordinates between transmitted and reflected light; the values were obtained using the formula:

OP = [(a*_b_ − a*_w_)^2^ +(b*_b_ − b*_w_)^2^]^1/2^ (4)

CR = Y_b_/Y_w_ = [(L* + 16)/116]^3^ × 100 (5)

CR values may range from 0 (totally transparent) to 1 (totally opaque).

The total colour change value ΔE* was achieved using the Formula (6).

ΔE* = [(ΔL*)^2^ + (Δa*)^2^ + (Δb*)^2^]^1/2^ (6)

The recordings were performed for each group in each stage.

To report the color change to a clinical standard, ΔE* was converted to NBS units according to the formula: NBS = ΔE* × 0.92 [24,25].

In conformity with NBS, the levels of color changes (expressed in NBS units) were: trace (0.0–0.5), slight (0.5–1.5), perceivable (1.5–3.0), marked (3.0–6.0), extremely marked (6.0–12.0), and change to another color (>12.0) [26]. 

### 2.6. Statistical Analyses

All measured and calculated data were expressed as means ± standard deviations. Data for each stage were analyzed using two-way ANOVA and paired Student’s *t*-test to assess differences between materials. Unpaired Student’s *t*-test was used to compare the values before and after immersion and desiccation for each material. Post hoc tests were conducted as multiple comparation methods for pairwise combinations. Bonferroni corrections were used in order to control the increased risk of type I errors resulting from multiple comparisons. All statistical analyses were performed using statistical software IBM SPSS Statistics software (IBM, New York, NY, USA). Pearson correlation and regression analyses were performed to evaluate the interdependence between the variables recorded in different stages. The significance of the Pearson coefficient (r) was related to 0–0.2 (very weak), 0.2–0.4 (weak), 0.4–0.6 (moderate), 0.6–0.8 (strong), and 0.8–1.0 (very strong). The coefficient of determinations (r^2^) indicated the percentage of the total variation of the dependent variable (L*, a*, b*, TP, OP) that is explained by the variation of the independent variable (material, stage). A significance level of 0.05 was set. For Bonferroni corrections, the alpha (α) level for a family of statistical tests was adjusted to new alpha.

## 3. Results

**Figure 1:**
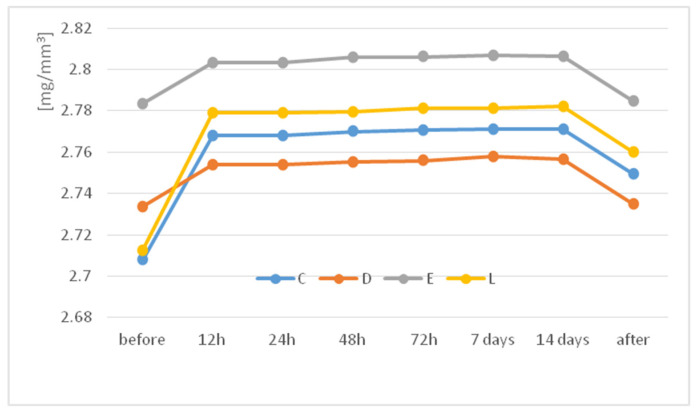
Time-dependent water absorption behavior (up to 2 weeks) of the thermoplastic materials.

Figure 1 displays the time-dependent water sorption behavior of the investigated materials.

A sudden increase of water sorption within the first 12 h of water exposure can be observed before approaching the saturation level. The calculated water sorption was between 2.15 and 2.19%, and no water solubility was registered. Related to the materials, the water uptake for E was significantly higher than for C (*p* = 0.094) and D (*p* = 0.009). Water uptake significantly increases the specific weight of the materials (*p* = 0.038), and after desiccation, the mass decrease is also significant (*p* = 0.000).

**Figure 2:**
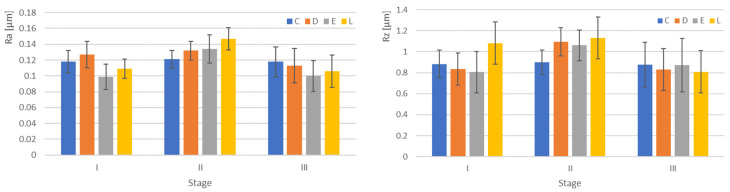
Graphical representation of the microroughness mean values and SD values for all materials related to each stage (I dryed, II after water immersion, III re-dreyed).

The mean Ra and Rz values with the standard deviations are shown in Figure 2 and Table 1. Significant differences were recorded in the first and second stage between a part of the materials and between the first and second stage for two materials (Table 2). Post hoc tests revealed these significant differences for pairwise combinations. For the Bonferroni correction, the alpha (α) level for a family of statistical tests was adjusted so that we control for the probability of committing a type I error. The new α was 0.05/6 = 0.0083, related to the 4 groups taken into the study. The Pearson coefficient r = 0.67 indicates a strong correlation between Ra and Rz values.

**Figure 3:**
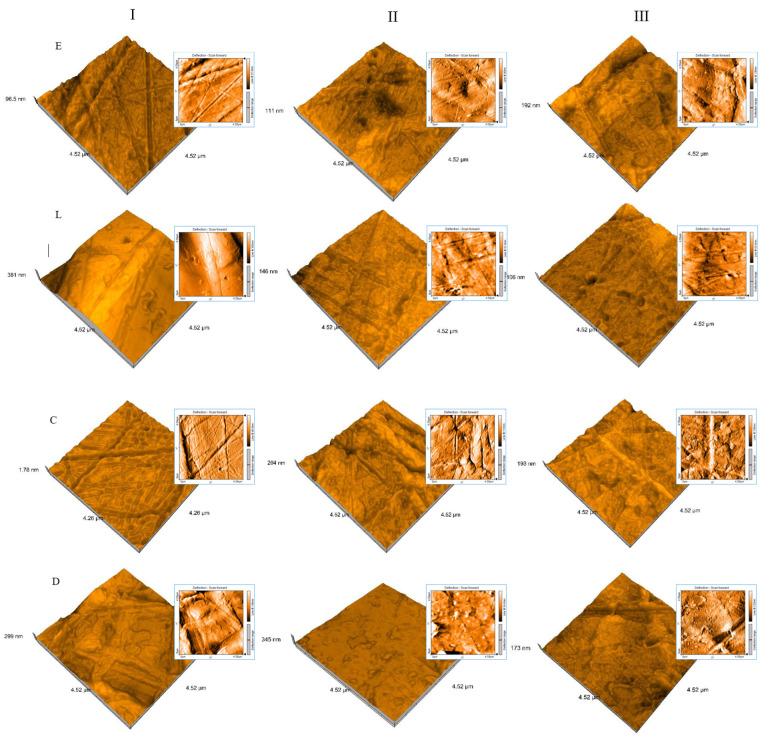
AFM 3D images of 4 × 4 μm^2^ scan areas for all materials (Crystal^®^ [C], Duran^®^ [D], Erkodur [E], and Leone [L]) related to each stage.

The shape and surface roughness of different samples were measured from AFM images. To visualize the difference in topography of the materials in different stages, AFM 3D images of scan areas of size 4 × 4 μm^2^ are shown in Figure 3.

**Figure 4:**
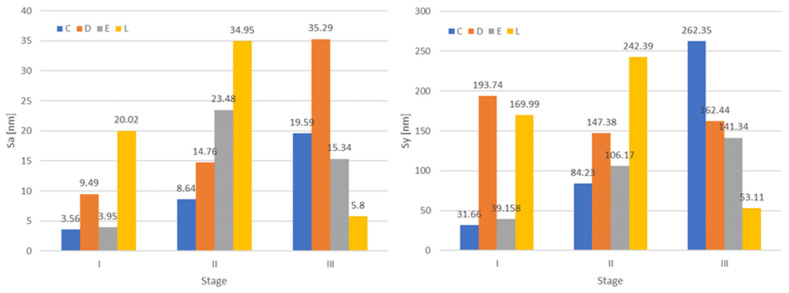
Graphical representation of the nano-roughness values for all materials related to each stage (I dryed, II after water immersion, III re-dreyed).

The surface roughness is due to the inhomogeneity in the network structure of particles. The roughness value increased with the water uptake and decreased after desiccation, remaining still larger than initially (Figure 4).

**Figure 5:**
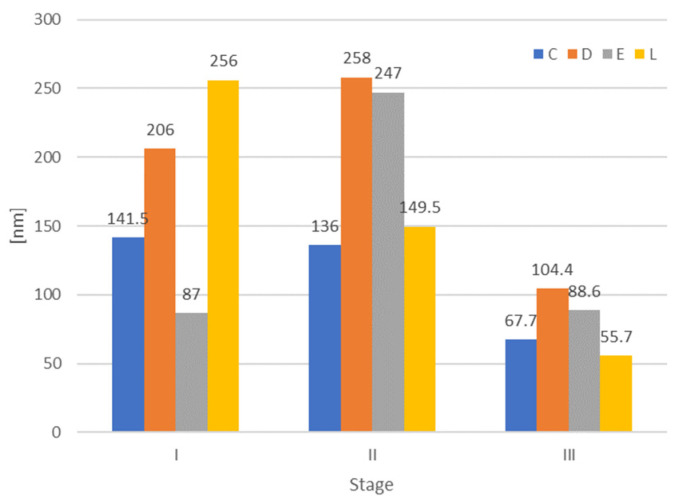
Measured particle size, for all materials, related to each stage (I dryed, II after water immersion, III re-dreyed).

The particle size is determined from the Z-height of the extract profile. The average particle size of the samples is shown in Figure 5.

In terms of nano-roughness (Sa and Sy), statistical analyses did not highlight significant differences neither between materials nor between stages (water uptake or desiccation). Related to the particle size, a significant decrease was registered after desiccation (*p* = 0.012). The Pearson coefficient r = 0.67 indicates a strong correlation between Sa and Sy values.

**Figure 6:**
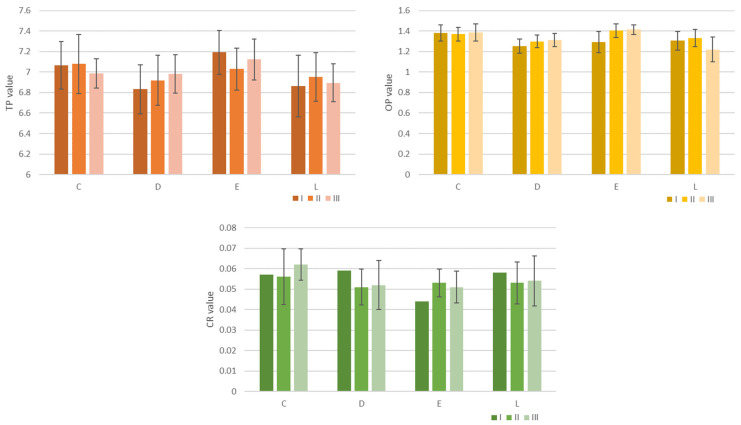
Graphical representation of the optical parameters: translucency (TP), opalescence (OP), and contrast ratio (CR)—mean values of and SD values for all materials (Crystal^®^ [C], Duran^®^ [D], Erkodur [E], and Leone [L]) related to each stage.

For the optical analyses Figure 6 and Table 3, Table 4 and Table 5 display, the calculated optical parameters were TP, OP, and CR. Post hoc tests considering Bonferroni corrections revealed no significant differences for pairwise combinations. For TP, OP, and CR, no significant differences were found between materials or between stages.

Results of the regression analyses are displayed in Table 6 for all the optical parameters and for all materials related to the optical coordinates L*, a*, b*.

Positive regression coefficients were calculated, which indicate that as the value of the optical parameters TP and OP increase, the variable L*, a*, b* also tend to increase. When the variable is statistically significant (bold characters), a worthwhile addition to the regression model is stated. Strong and very strong correlations that are statistically significant are between TP and a* for all materials, between TP and b* for D, and between OP and b* for all materials. The larger the coefficient of determination R2, the better the regression model fits.

Related to color changes ΔE* converted to NBS units, for the most part, slight changes were registered both after hydration and after desiccation (very slight only for D after hydration) (Table 7).

## 4. Discussion

Previous studies indicated that water sorption accelerated the stress relaxation of the matrix of thermoplastic materials by plasticization. This may induce a split of the intrachain and/or interchain bonds, alter the free volume of the polymers, and wash out soluble components, which speeds up the degradation process [2,27,28,29,30]. Water uptake is often accompanied by swelling and, hence, dimensional changes in the appliances. Therefore, an ideal thermoplastic material for orthodontic purposes should have a water sorption as low as possible [11,28,31].

Because PET-G is a thermoplastic material with highly viscoelastic behavior, the stress induced by deformations tends to determine the irreversible deterioration of the mechanical properties. This phenomenon, called stress relaxation, results in additional alteration of the ability to be used for orthodontic appliances. Therefore the stress relaxation of thermoplastic materials within the oral environment is crucial for a better understanding the biomechanics of the appliances [2,10,32].

The optical properties, the strength, and elastic modulus of thermoplastic materials decrease after thermoforming, and water sorption increases. The materials are deformed and decrease in thickness. The thickness of the processed materials decreased from 1 mm to 0.68–0.75 mm. The crystallinity is altered during thermoforming, which has an effect on the mechanical and optical properties. Therefore, previous studies show the translucency, water sorption and solubility, surface hardness, and elasticity may be altered during thermoforming [11].

Several studies investigated the effect of different environmental conditions on the mechanical behavior of thermoplastic materials. They related the mechanical properties to environmental factors and the molecular structure of the materials [12]. This study investigated the time-dependent water sorption behavior of different PET-G thermoplastic materials. Related to the materials, the water uptake for E was significantly higher than for C and D. For all, a steep increase in water sorption within the first 12 h of water exposure was observed before approaching the saturation level. The calculated water sorption was 2.15–2.19%, with a significant increase in the specific weight.

Other studies reported that the water absorption of PET-G increased to 0.8% in their 2-week experiment [22]. These polymers show a steep increase in water absorption within the first 24 h of water exposure before approaching a saturation level, and the saturation level depends on the respective polymer.

Understanding the origins of roughness is of great interest for thermoplastic appliances, as it directly influences key mechanical, chemical, and optical properties of the materials [33]. Surface roughness is an important factor for the aesthetic, technical, and biological success. The risk for crack initiation and propagation may be higher when the surface is rougher. Surface roughness has an influence on bacterial adhesion, and it is postulated that the critical mean surface roughness for increased bacterial adhesion is 0.2 μm [34]. Recorded mean values of the arithmetic average roughness Ra are between 0.09 and 0.14 μm. Due to the fact that the maximum absolute vertical roughness (Rz) may better explain the surface properties, it was also taken into consideration. Values were in the interval 0.806–1.132 μm and strongly correlated to Ra (r = 0.67).

Multiscale roughness analyses were performed for the topography of the surfaces because it stretches from the micron size of the components down to the atomic size. Roughness at larger or smaller scales may play different roles depending on the context. The thermoplastic materials surfaces topography influences their clinical long-time performance because it is related to the key mechanical, tribological, chemical, and optical properties. More precisely, surface evaluation can be used to extrapolate their future behavior in the oral environment. The functional properties, durability, and optical characteristics are governed by the surface roughness parameters.

To visualize the shape and surface topography of the materials in different stages, AFM 3D images were recorded, and nano-roughness was measured. Values increased with the water uptake and decreased after desiccation, remaining still larger than initially. The Pearson coefficient r = 0.67 indicates a strong correlation between Sa and Sy values. Related to the particle size, a significant decrease was registered at desiccation. The first hypothesis was accepted.

Because aesthetics play an important role in the acceptance of appliances, their optical properties are expected to be preserved. However, the transparency and color stability of clear appliances are known to be affected by water sorption [10,14].

Optical parameters TP, OP, and CR were obtained. For TP, OP, and CR, no significant differences were found between materials or between stages. Regression analyses were developed for all the optical parameters for all materials related to the optical coordinates L*, a*, b* in order to obtain a regression model. Statistically significant strong and very strong positive correlations were observed between TP and a* for all materials, between TP and b* for D, and between OP and b* for all materials. Related to the color, slight changes were registered. The second hypothesis was accepted as well.

The third hypothesis, which was related to the interaction between the materials taken into the study and the stage related to water sorption, was partially accepted. Significant differences in terms of microroughness were found between a part of the materials, D/I-E/I, C/II-L/II, and for two of them (E and L) between the first and second stage.

This study has some limitations because flat specimens were taken into consideration in order to achieve surface and optical characteristics. Intraoral conditions related to water sorption were only partially taken into consideration; the temperature was maintained constant during the whole study. Temperature and pH variations should be addressed in future studies. We are aware of the importance of reproducing a complex simulated oral environmental behavior. The surface topography (both on micron- and nano-scale) contributes substantially. It may be quite an ambitious endeavor not only to separately evaluate each parameter, but also their interaction. Immediate future challenges related to the behavior of clear thermoplastic appliances would be related to the interactions of different environmental factors on their surface characteristics and aesthetics. Likewise, including other classes of thermoplastic materials would increase the vision of their simulated oral behavior.

## 5. Conclusions

On the basis of the investigations and their limitations, the following can be concluded:

The specific weight of the studied PETG materials significantly increases within the first 12 h of water exposure, even if the studied materials have a different behavior.

Arithmetic average roughness values increased with the water uptake, but they continue to be kept below the critical mean surface roughness. On nano-level, 3D images show an increase in inhomogeneity in the network structure of particles.

Related to the optical properties, regression models indicated positive correlations between TP and a* and between OP and b* with water uptake. Besides this, slight color changes were registered.

## Figures and Tables

**Table 1:** Mean values of microroughness and SD values for all materials related to each stage.

Material/ Stage	I		II		III	
	Ra	Rz	Ra	Rz	Ra	Rz
C	0.118 ± 0.014	0.883 ± 0.131	0.121 ± 0.011	0.902 ± 0.116	0.118 ± 0.019	0.877 ± 0.212
D	0.127 ± 0.017	0.835 ± 0.153	0.132 ± 0.012	1.094 ± 0.136	0.113 ± 0.022	0.829 ± 0.199
E	0.099 ± 0.016	0.806 ± 0.196	0.134 ± 0.018	1.061 ± 0.146	0.100 ± 0.019	0.871 ± 0.254
L	0.109 ± 0.012	1.083 ± 0.112	0.147 ± 0.014	1.132 ± 0.155	0.106 ± 0.021	0.809 ± 0.218

**Table 2:** *p*-values of significant group differences for Ra values.

Compared Groups: Material/Stage	*p* Value
D/I-E/I	0.001
C/II-L/II	0.000
E/I-E/II	0.000
L/I-L/II	0.000

**Table 3:** Mean values and SD values of the translucency parameter (TP) for all materials related to each stage.

Material/ Stage	TP		
	I	II	III
C	7.068 ± 0.231	7.079 ± 0.288	6.986 ± 0.142
D	6.832 ± 0.237	6.920 ± 0.246	6.983 ± 0.186
E	7.192 ± 0.215	7.030 ± 0.203	7.123 ± 0.198
L	6.865 ± 0.300	6.952 ± 0.238	6.894 ± 0.185

**Table 4:** Mean values of and SD values of the opalescence parameter (OP) for all materials related to each stage.

Material/ Stage	OP		
	I	II	III
C	1.379 ± 0.078	1.379 ± 0.066	1.379 ± 0.083
D	1.252 ± 0.069	1.252 ± 0.063	1.252 ± 0.062
E	1.293 ± 0.102	1.293 ± 0.067	1.293 ± 0.047
L	1.305 ± 0.092	1.305 ± 0.082	1.305 ± 0.120

**Table 5:** Mean values of and SD values of the contrast ratio (CR) for all materials related to each stage.

Material/ Stage	CR		
	I	II	III
C	0.057 ± 0.009	0.057 ± 0.009	0.057 ± 0.009
D	0.059 ± 0.007	0.059 ± 0.007	0.059 ± 0.007
E	0.044 ± 0.006	0.044 ± 0.006	0.044 ± 0.006
L	0.058 ± 0.013	0.058 ± 0.013	0.058 ± 0.013

**Table 6:** Regression analysis of the optical parameters for all materials related to the optical coordinates L*, a*, b*.

Optical Parameter/ Material	L*	a*	b*
TP/C	moderate, r = 0.524 r^2^ = 0.275 *p* = 0.867	**very strong, r = 0.822** **r^2^ = 0.676** ***p* = 0.002**	moderate, r = 0.531 r^2^ = 0.282 *p* = 0.096
TP/D	strong, r = 0.654 r^2^ = 0.428 *p* = 0.546	**very strong, r = 0.998** **r^2^ = 0.997** ***p* = 0.000**	**very strong, r = 0.995** **r^2^ = 0.991** ***p* = 0.028**
TP/E	strong, r = 0.613 r^2^ = 0.375 *p* = 0.703	**strong, r = 0.623** **r^2^ = 0.388** ***p* = 0.004**	very strong, r = 0.873 r^2^ = 0.763 *p* = 0.122
TP/L	strong, r = 0.673 r^2^ = 0.453 *p* = 0.472	**very strong, r = 0.944** **r^2^ = 0.892** ***p* = 0.001**	moderate, r = 0.500 r^2^ = 0.250 *p* = 0.071
OP/C, D, E, L	strong, r = 0.782 r^2^ = 0.612 *p* = 0.700	weak, r = 0.386 r^2^ = 0.149 *p* = 0.000	**strong, r = 0.736** **r^2^ = 0.542** ***p* = 0.042**

**Table 7:** Color changes ΔE* converted to NBS units after hydration and after desiccation.

Material/ Period	ΔE* NBS	
	After Water Uptake	After Desiccation
C	0.540	0.540
D	0.386	0.386
E	0.745	0.745
L	0.581	0.581

## Data Availability

Not applicable.
